# Linear dependence of surface expansion speed on initial plasma temperature in warm dense matter

**DOI:** 10.1038/srep29441

**Published:** 2016-07-12

**Authors:** W. Bang, B. J. Albright, P. A. Bradley, E. L. Vold, J. C. Boettger, J. C. Fernández

**Affiliations:** 1 Los Alamos National Laboratory, Los Alamos, New Mexico 87545, USA

## Abstract

Recent progress in laser-driven quasi-monoenergetic ion beams enabled the production of uniformly heated warm dense matter. Matter heated rapidly with this technique is under extreme temperatures and pressures, and promptly expands outward. While the expansion speed of an ideal plasma is known to have a square-root dependence on temperature, computer simulations presented here show a linear dependence of expansion speed on initial plasma temperature in the warm dense matter regime. The expansion of uniformly heated 1–100 eV solid density gold foils was modeled with the RAGE radiation-hydrodynamics code, and the average surface expansion speed was found to increase linearly with temperature. The origin of this linear dependence is explained by comparing predictions from the SESAME equation-of-state tables with those from the ideal gas equation-of-state. These simulations offer useful insight into the expansion of warm dense matter and motivate the application of optical shadowgraphy for temperature measurement.

Rapid heating of matter with intense laser beams^1^,^2^,^3^,^4^,^5^,^6^, strong x-ray sources^7^,^8^, laser-driven ion sources^9^,^10^,^11^,^12^,^13^,^14^, and compression using lasers^15^,^16^,^17^,^18^ or pulsed-power machines^19^,^20^ has enabled the study of near-solid-density materials in extreme temperatures (1–100 eV, or 10^4^–10^6^ K) and/or pressures (≥10^11^ Pa). Materials in these extreme states, or in the warm dense matter^21^ regime, are frequently found in planetary interiors^16^,^18^ and in high energy density physics experiments^8^,^15^,^22^,^23^,^24^, but their properties are difficult to predict theoretically^25^,^26^ and are not well known. Experimental validation of existing theoretical models in this regime is also very challenging because of the difficulty of interpretation and measurement of warm dense matter properties. Only very recently has the production of uniformly heated warm dense matter samples been demonstrated in the laboratory^27^,^28^,^29^ and such homogeneous samples are expected to be useful for conductivity^1^,^4^, opacity^7^, equation-of-state (EOS)^11^,^12^,^15^, stopping power^24^, and resistivity^30^ measurements.

When a sample is heated rapidly to the warm dense matter regime, it promptly starts expanding outward. The expansion of warm dense plasmas was visualized with an optical streak camera in our recent experiment^27^, where we noted that the expansion speeds were directly related to the initial temperatures of the samples immediately after heating. In this Article, we examine the time-averaged surface expansion speed of a solid-density gold foil in the warm dense matter regime using the RAGE radiation-hydrodynamics code^31^. Contrary to the common belief that the expansion speed of a warm dense plasma will be proportional to 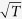, where *T* is the plasma temperature, we find that the average expansion speed increases almost linearly with the initial temperature of the gold foil in this regime. We follow the motion of three surfaces that correspond to the critical electron density surfaces for 660, 500, and 400 nm optical light in two-dimensional (2D) RAGE simulations, and all show the same trend. We find that this behavior can be explained by comparing predictions from the SESAME EOS tables for gold^32^,^33^ with those from the ideal gas EOS.

## Results

Recent experiments^27^,^28^ on the Trident laser facility at Los Alamos National Laboratory (LANL) demonstrated the production of uniformly heated 10 μm thick warm dense gold foils. The Trident laser delivered 60–80 J, 650 fs, 1054 nm wavelength pulses to irradiate 110 nm thick aluminum foils with a peak laser intensity of about 2 × 10^20^ W/cm^2^ and successfully generated quasi-monoenergetic aluminum ion beams^10^. The laser-driven ion beams diverged with a 20° cone half-angle^10^ and irradiated gold foils located 2.37 mm from the ion sources inside the vacuum target chamber. The gold foils were heated uniformly and rapidly (~20 ps) to 5–6 eV by the aluminum ions and expanded into vacuum^27^,^28^. In ref. 27, an optical probe beam (660 nm wavelength) was used to measure the surface expansion speed with optical shadowgraphy and the initial temperature of warm dense gold was inferred using the one-to-one relationship between the optical expansion speed and the initial temperature.

**Figure 1:**
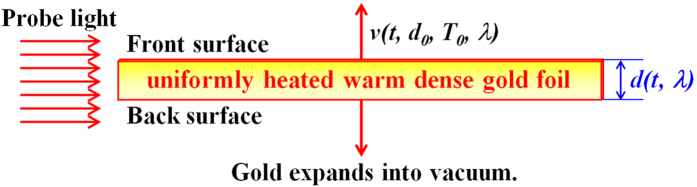
Schematic drawing of a uniformly heated warm dense gold foil that expands into vacuum. A hypothetical optical probe light of wavelength *λ* is incident from the side, and defines the front and the back surfaces of the gold foil as the foil expands in time. The thickness of the foil is defined as the distance between the two critical electron density surfaces of the foil, and increases in time. Both the front and the back surfaces expand into vacuum at the same speed of *v*(*t, d*_*0*_*, T*_*0*_*, λ*).

Figure 1 shows a schematic drawing of an expanding warm dense gold foil at time *t* with an expansion speed *v*(*t, d*_*0*_*, T*_*0*_*, λ*), where *d*_*0*_ is the initial thickness of the foil, *T*_*0*_ is the initial temperature of the warm dense gold foil after heating, and *λ* is the wavelength of the probe beam for optical shadowgraphy. A hypothetical optical probe (red arrows in the figure) of wavelength *λ* is incident from the side to image the front and the back surfaces of the foil as a function of time *t*. We define the front and the back surfaces of the foil as the locations of the two critical electron density surfaces for the hypothetical probe light of wavelength *λ*. The thickness of the foil, *d*(*t, λ*), is defined as the distance between the two critical electron density surfaces of the foil, and increases in time as the foil expands into vacuum. The measured thickness of the foil depends on the wavelength of the probe light because the critical electron density is wavelength-dependent. The surface expansion speed is written as *v*(*t, d*_*0*_*, T*_*0*_*, λ*) to indicate explicitly that the speed changes in time and depends on the initial thickness and temperature of the foil and the wavelength of the probe light. The foil is wide (>100 μm) in the horizontal direction in our 2D RAGE simulations.

**Figure 2:**
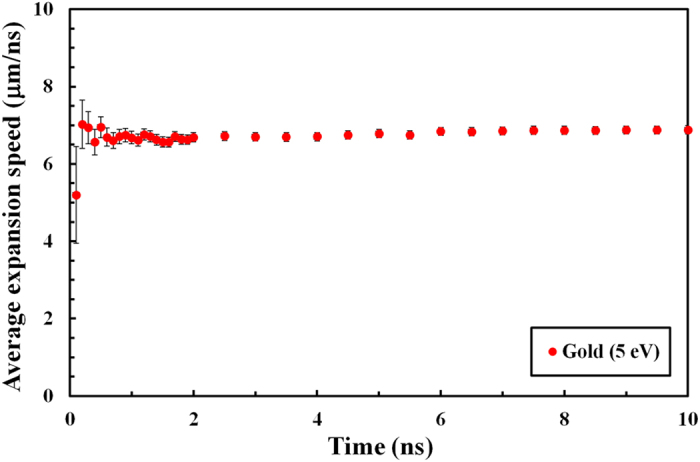
Average expansion speed as a function of time. The average surface expansion speed from a 2D RAGE simulation is shown as a function of time from 0 to 10 ns for a 660 nm probe light. The initial temperature of the gold foil is 5 eV, and the foil thickness is 10 μm in the simulation. The error bars are estimated from the simulation mesh size.

In Fig. 2, the average expansion speed of a 5 eV gold foil from a 2D RAGE simulation is shown from 0 to 10 ns. RAGE is a multi-dimensional, adaptive-mesh-refinement, Eulerian, radiation-hydrodynamics code^31^. Currently, the RAGE code does not have the capability to simulate ion-beam energy deposition, so to mimic it we used a spatially homogeneous 2D energy source that is Gaussian in time with a 20 ps full-width-at-half-maximum. We created electron density maps from RAGE output and recorded the location of the critical density surface for 660 nm light. The time-averaged expansion speed of gold in Fig. 2 is calculated as


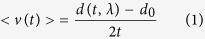


where *λ* = 660 nm and *d*_*0*_ = 10 μm in this simulation. The difference in the thickness is divided by 2 because both the front and the back surfaces expand into vacuum. The average surface expansion speed remains nearly unchanged after the first few hundred picoseconds in Fig. 2. The vertical error bars are estimated from the simulated image mesh size of 0.125 μm and from the fluctuations in the average expansion speeds of 0.1 μm/ns. The horizontal error bars are estimated as 0.05 ns from the time step of 0.1 ns.

**Figure 3:**
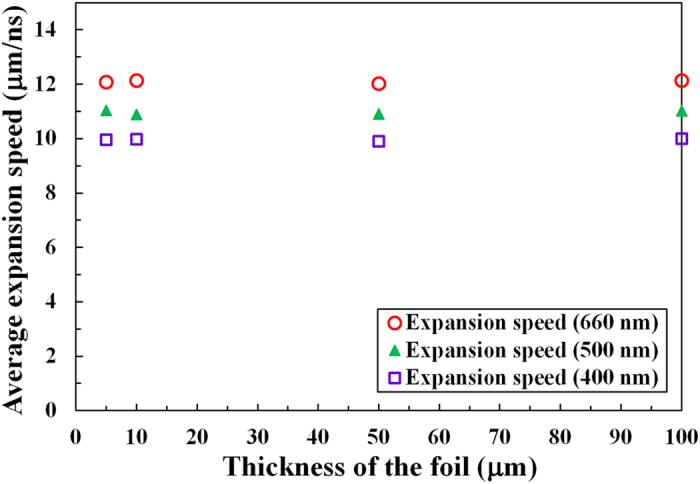
Average expansion speeds from RAGE simulations for different foil thicknesses. The average expansion speed of the foil surface is shown as a function of the foil thickness from 5 to 100 μm for three different wavelengths. The locations of the critical density surfaces for 660 nm, 500 nm, and 400 nm light are examined as functions of time in these simulations.

The average expansion speeds from RAGE simulations are shown for different initial foil thicknesses from 5 to 100 μm in Fig. 3. Hollow red circles show the expansion speeds of the critical electron density surfaces for 660 nm light, and solid green triangles indicate the expansion speeds of the critical density surfaces for 500 nm light. Hollow violet squares represent the expansion speeds of the critical density surface for 400 nm light. The initial temperature of the warm dense gold foil is 10 eV, and the surface expansion speed is averaged over the first two nanoseconds. We estimate the vertical error bars to be 0.1 μm/ns from fluctuations in the average expansion speeds; these error bars are too small to show in the figure.

Comparing electron number density profiles of the expanding gold foil at different times, we find that the expansion speed is not the same for all density levels. Depending on the density level to which a particular diagnostic is sensitive, there is a unique expansion speed associated with that density level^34^. We examine the expansion speeds for three different electron density surfaces in Fig. 3 to check the dependence of the expansion speed on the wavelength of the probe beam. In all three cases, RAGE simulations indicate that the expansion speed is predicted to be insensitive to the initial thickness of the gold foil. The critical density surface for 660 nm light (2.56 × 10^21^ electrons/cm^3^) expands at 12.1 (±0.1) μm/ns regardless of foil thickness, while the critical density surface for 500 nm light (4.46 × 10^21^ electrons/cm^3^) expands at a slightly slower speed of 11.0 (±0.1) μm/ns owing to the higher electron density. At the highest electron density, the critical density surface for 400 nm light (6.97 × 10^21^ electrons/cm^3^) expands at the slowest speed of 10.0 (±0.1) μm/ns for all foil thicknesses.

**Figure 4:**
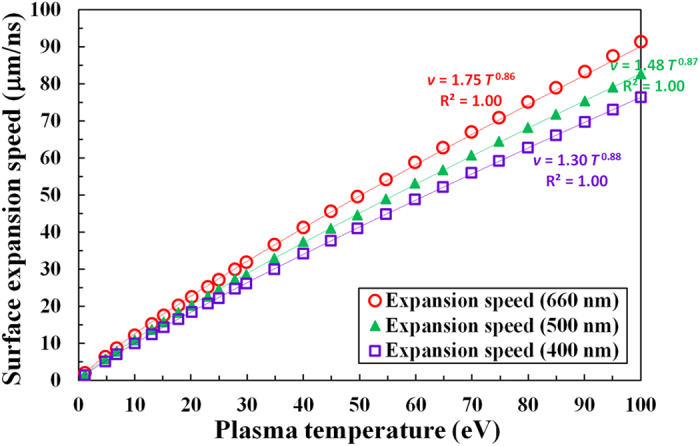
Nearly linear dependence of the surface expansion speed on the gold temperature in the warm dense matter regime. Surface expansion speeds from RAGE 2D simulations are shown for three wavelengths over plasma temperatures from 1 to 100 eV. The locations of the critical density surfaces for 660 nm, 500 nm, and 400 nm light are examined as functions of the initial temperature of warm dense gold in these simulations.

In Fig. 4, we show the relationship predicted from RAGE simulations between the surface expansion speed and the initial temperature of the gold foil for three different wavelengths in the warm dense matter regime (1–100 eV). The initial thickness of the gold foil is 10 μm, and the surface expansion speed is averaged over the first two nanoseconds. The vertical error bars are estimated from the fluctuations in the average expansion speeds, and are too small to show in the figure.

We have added power law fits in the figure and the exponents range from 0.86 to 0.88. The power law fit (solid red line) to the 660 nm data indicates that the expansion speed can be approximated as 1.75 × *T*[eV]^0.86^ [μm/ns] in this regime. Likewise, the power law fit (solid green line) to the 500 nm data indicates that the expansion speed can be estimated as 1.48 × *T*[eV]^0.87^ [μm/ns], while the fit (solid violet line) for the 400 nm data indicates that the expansion speed is 1.30 × *T*[eV]^0.88^ [μm/ns] in this regime. In all three cases, the power law fits represent the data very well with the coefficient of determination, denoted *R*^*2*^, greater than 0.999. Supplementary Fig. S3 confirms similar power law fits for 50 μm thick and 100 μm thick gold foils under the assumption of a similar uniform heat source. When straight lines are used to fit the data, we get an *R*^*2*^ value of 0.99 for all three cases, showing that a simple linear dependence is an excellent approximation.

**Figure 5:**
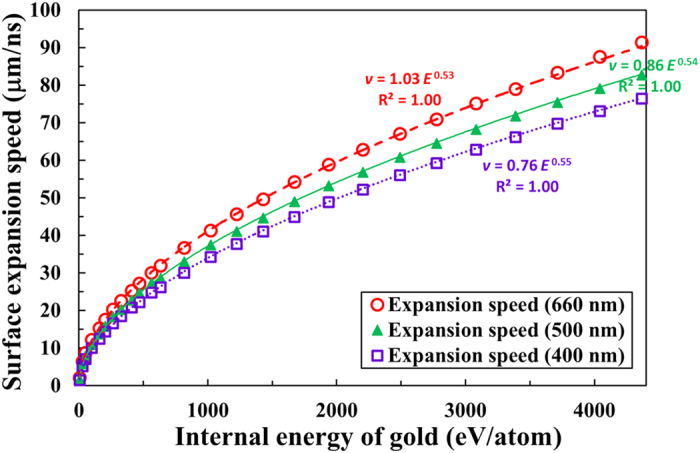
Expansion speed as a function of the internal energy of warm dense gold. Surface expansion speeds seen in Fig. 4 as a function of temperature are shown here as functions of the internal energy of gold atom (corresponding to a temperature range of 1 to 100 eV). The dashed red curve indicates the power law fit to the 660 nm data, while the solid green curve indicates the power law fit to the 500 nm data. The dotted violet curve shows the power law fit to the 400 nm data.

To understand this nearly linear dependence, we have examined the relationship between the expansion speed and the internal energy of gold in Fig. 5. The internal energy of a gold atom, or the absorbed energy per gold atom, is shown in units of eV/atom instead of the MJ/kg unit used in the SESAME library to represent its meaning clearly. An internal energy of 4400 eV/atom corresponds to a temperature of about 100 eV for gold. The initial thickness of the gold foil is 10 μm, and the expansion speed is averaged over the first two nanoseconds. Again, we have added power law fits in the figure and the exponents are very close to the expected value of 0.5 ranging from 0.53 to 0.55.

In contrast to Fig. 4 where the surface expansion speed increases almost linearly with the temperature of the gold, Fig. 5 confirms that the average expansion speeds follow 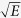 dependence in all three cases, with *E* representing the internal energy of a gold atom. The dashed red curve for the 660 nm data exhibits an *E*^0.53^ dependence and the solid green curve for the 500 nm data indicates an *E*^0.54^ dependence. The dotted violet curve for the 400 nm data also shows a similar dependence on the internal energy, *E*^0.55^. The observed ~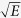 dependence is expected because the absorbed energy eventually turns into the collective outward motion of the ions and electrons and their kinetic energy increases quadratically with their expansion speed.

**Figure 6:**
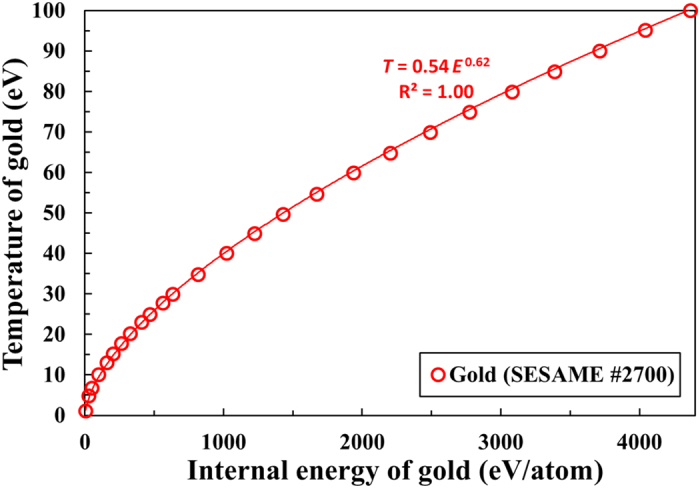
Temperature of gold as a function of the internal energy in the warm dense matter regime. The temperature of gold from RAGE simulations is plotted as a function of the internal energy of gold atom from 4 to 4400 eV/atom. The solid red curve shows the power law fit to the data.

Comparing Figs 4 and 5, we infer that the temperature of warm dense gold does not increase linearly with the internal energy. The relationship between the temperature of solid density gold and the internal energy in the warm dense matter regime is shown explicitly in Fig. 6. The hollow red circles indicate the temperature of gold from our RAGE simulations, which is plotted as a function of the internal energy from 4 to 4400 eV/atom (corresponding to temperatures ranging from 1 to 100 eV). The power law fit (solid red curve) to the data indicates that the temperature of gold is proportional to *E*^0.62^ in this regime. It is obvious from the figure and the power law fit that the temperature does not increase linearly with the internal energy of gold in this regime. (Note that the ideal gas EOS, written as *E* = 3*NkT*/2, where *N* is the number of particles and *k* is the Boltzmann constant, implies a linear dependence. In this Article, *kT* is defined as the temperature *T*[eV].)

The power law fit in Fig. 6 indicates that the temperature of gold can be approximated as *T*[eV] = 0.54*E*[eV/atom]^0.62^ in the warm dense matter regime. This can be used in the power law fits in Fig. 5 to find the relationship between the expansion speed and the temperature. For example, the expansion speed is approximated as 1.03*E*[eV/atom]^0.53^ in Fig. 5 for 660 nm light and this is equivalent to stating that the expansion speed can be written as


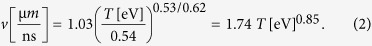


Equation (2) is practically the same as the power law fit for the 660 nm data shown in Fig. 4. Indeed, we recover the linear dependence between the expansion speed and the temperature using Figs 5 and 6 and confirm that the observed linearity in Fig. 4 can be explained using the non-ideal gas behavior illustrated in Fig. 6.

**Figure 7:**
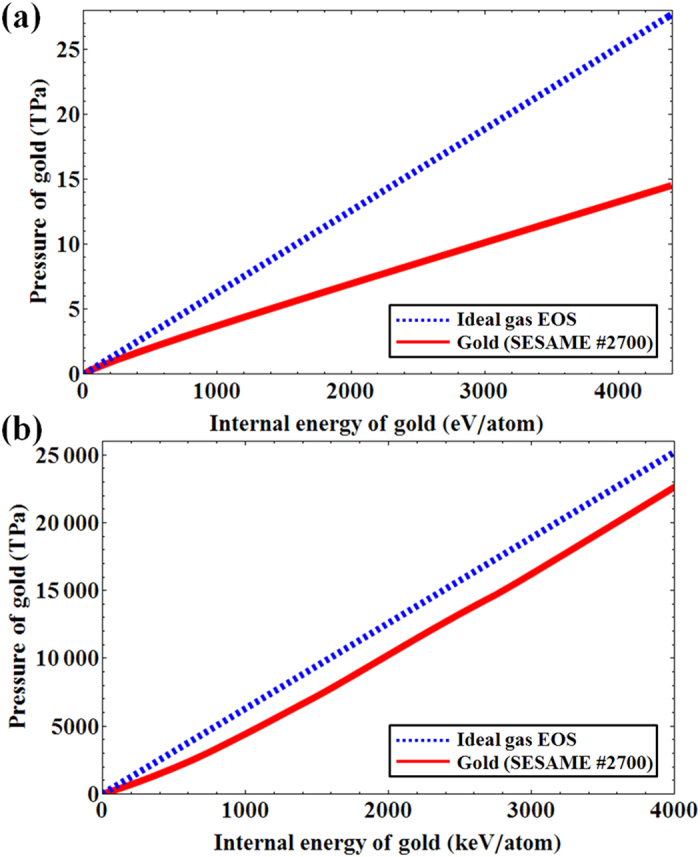
Differences between the ideal gas EOS and the SESAME #2700 table. (**a**) Pressure of gold is shown as a function of the internal energy of gold atom from 0 to 4400 eV/atom. The dashed blue line indicates the prediction from the ideal gas EOS, whereas the solid red line shows the expected pressure of gold from the SESAME #2700 table. In the warm dense matter regime shown in this figure, the two predictions differ significantly. (**b**) Pressure of gold is shown as a function of the internal energy of gold atom from 0 to 4000 keV/atom. The dashed blue line indicates the prediction from the ideal gas EOS, whereas the solid red line shows the expected pressure of gold from the SESAME #2700 table. As expected for high temperature plasmas, the predictions from the SESAME #2700 table are close to those from the ideal gas EOS.

To highlight the difference between the SESAME EOS tables and the ideal gas EOS in the warm dense matter and in the high-temperature regimes, we plot the predicted pressures of gold as functions of the internal energy in Fig. 7(a,b). In both figures, the dashed blue lines indicate the pressures predicted from the ideal gas EOS, whereas the solid red lines show the pressures expected from the SESAME EOS #2700 table. Figure 7(a) shows the pressure of solid density gold in the warm dense matter regime from 1 to 100 eV, while Fig. 7(b) shows the pressure of gold in the high temperature plasma regime up to 30 keV (4000 keV/atom corresponds to 30 keV for gold according to the SESAME EOS #2700 table).

The total pressure of gold from the ideal gas EOS is calculated as


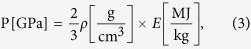


where *ρ* = 19.3 g/cm^3^ is the density of gold and the internal energy is written in MJ/kg unit so that the coefficients become simpler. The pressures from the SESAME EOS #2700 table in Fig. 7(a) show clear deviations from the ideal gas EOS in the warm dense matter regime. In contrast, the expected pressures in Fig. 7(b) (corresponding to a temperature range of 0 to 30 keV) indicates that the differences are much smaller in the high temperature plasma regime. We have not included the predicted pressures from the SESAME EOS #2705 table because it is known to be valid only up to 0.8 TPa^33^.

## Discussion

We have investigated the predicted relationship between the expansion speed of a uniformly heated warm dense gold foil target and the initial temperature of the gold. The expansion speed of the gold, according to RAGE simulations, increases almost linearly with temperature in all three cases. We have shown that this near linear dependence can be understood from the nonlinear dependence between the temperature and the internal energy of gold.

In our simulations, the average surface expansion speed of warm dense gold remains nearly constant after the first few hundred picoseconds. The constant expansion speed after the initial acceleration stage is expected because of the work-free nature of the expansion. There is practically no more push from the bulk warm dense gold after the foil has expanded for several hundred picoseconds, and the gold atoms expand freely into vacuum without gaining or losing their kinetic energies. Indeed, Fig. 2 presents no signs of increase or decrease in the expansion speed after the first few hundred picoseconds.

In Fig. 3, RAGE simulations predict that the expansion speed is insensitive to the initial foil thicknesses. Since we have studied uniformly heated warm dense plasmas with no temperature gradients, there is a pressure balance everywhere within the plasma except at the surface. The pressure imbalance at the surface is responsible for the acceleration of gold atoms toward vacuum, and the gold atoms near the surface collectively move outward. This does not depend on the thickness of the foil because the bulk plasma does not contribute to the pressure imbalance at the surface.

RAGE simulations in Fig. 3 also predict that the expansion speed depends on the density level. This dependence could be explained at least qualitatively by looking at the solution to the Riemann problem for a free hydrodynamic expansion of a plasma into vacuum. In this solution, the tenuous front propagates faster than the dense fluid media behind. The velocity is a linearly increasing function of position and the density decays as the rarefaction front eats back into the fluid blob at a rate on the order of the sound speed. Supplementary Fig. S4 illustrates this point.

In Figs 4 and 5, we estimated the vertical error bars from the fluctuations in the average expansion speeds in our RAGE simulations. Although we believe this is a valid method to estimate the error bars, we acknowledge that there could be an additional systematic error of up to 13% in this regime because uncertainties in the SESAME EOS tables in the warm dense matter regime can be significant. For example, the SESAME EOS tables #2700 and #2705 exhibit a 13% difference in the predicted temperatures of gold near 3.5 eV. The differences become gradually smaller as temperature increases, but there is still a 6% difference expected at 10 eV. Since the SESAME #2705 table is valid only up to 0.8 TPa (10.5 eV for solid density gold), we have not attempted to estimate the systematic error bars.

The temperature of gold was found to increase nonlinearly with the internal energy as seen in Fig. 6. We postulate that this behavior is related to the increasing ionization with temperature. The charge state of gold increases continuously as temperature increases in this regime, and reaches <Z> ~15 ( = 15 free electrons per gold atom) at 100 eV. The temperature of gold cannot increase linearly with the internal energy because a substantial fraction (~50% for 100 eV gold)^35^ of the internal energy goes into ionization.

The predicted nonlinear increase of the plasma temperature with the internal energy appears to be a general characteristic of a partially ionized plasma such as warm dense matter. We have examined the dependence of plasma temperature on internal energy from 1 to 100 eV for other materials such as aluminum (SESAME EOS #3720 table, *T*[eV] = 0.50*E*[eV/atom]^0.70^) and copper (SESAME EOS #3336 table, *T*[eV] = 0.53*E*[eV/atom]^0.66^), and have found similar nonlinear relationships in this regime. Supplementary Fig. S1 shows the average surface expansion speed of a warm dense aluminum foil, which also predicts a nearly linear dependence between the expansion speed and the temperature.

The surface expansion speed of an ideal plasma expanding into vacuum is known to be proportional to the sound speed^34^ and we have calculated the sound speeds using the SESAME EOS tables in the warm dense matter regime. Supplementary Fig. S2 shows the calculated sound speed in solid density gold as a function of temperature from 1 to 100 eV. The sound speeds are found to increase almost linearly with the temperature of gold in this regime, which is consistent with the observed, nearly linear dependence of expansion speed in Fig. 4.

We believe these simulations offer useful insight into the expansion of warm dense matter and point to a straightforward method using optical shadowgraphy to obtain the initial temperature of a uniformly heated warm dense matter sample. For warm dense gold, we found the expansion speeds as functions of the initial plasma temperature for three different wavelengths. Interpolation could be used for optical shadowgraphy with an intermediate wavelength light.

## Methods

### Average expansion speeds from RAGE simulations

We have used the electron density maps created from 2D RAGE radiation-hydrodynamics simulations to calculate the average expansion speeds of three different electron density surfaces. Specifically, we recorded the locations of the critical density (*n*_*c*_[cm^−3^] = 1.115 × 10^21^/*λ*[μm]^2^) surfaces for 660 nm, 500 nm, and 400 nm light as functions of time from 0 to 2 ns with a time step of 0.1 ns. As seen in Fig. 2, which showed nearly constant average expansion speed after the first few hundred picoseconds from heating, the expansion speeds of gold averaged over the first 0.5, 1.0, and 2.0 ns were the same to within the mesh induced uncertainties. We used the time-averaged expansion speed of gold at 2.0 ns in Figs 3–5.

## Additional Information

**How to cite this article**: Bang, W. *et al*. Linear dependence of surface expansion speed on initial plasma temperature in warm dense matter. *Sci. Rep.* **6**, 29441; doi: 10.1038/srep29441 (2016).

## Supplementary Material

Supplementary Information
